# Noninfectious Uveitis in Rheumatology: Patterns, Treatment, and Outcomes

**DOI:** 10.7759/cureus.39965

**Published:** 2023-06-05

**Authors:** Raheel Younus, Muhammad A Saeed, Muhammad Arshad, Sumaira Farman, Nighat M Ahmad

**Affiliations:** 1 Rheumatology, National Hospital and Medical Centre, Lahore, PAK; 2 Rheumatology, Central Park Medical College, Lahore, PAK; 3 Rheumatolgy, University of Health Science, Lahore, PAK

**Keywords:** rheumatology & autoimmune diseases, disease-modifying antirheumatic drugs (dmards), biologic therapies, uveitis, idiopathic uveitis, systemic juvenile idiopathic arthritis, spondyloarthritis, non-infectious uveitis

## Abstract

Objectives: The present study aimed to determine the pattern and cause of noninfectious uveitis in rheumatology practice. The secondary objective was to identify the pattern of treatment and outcomes.

Materials and methods: This retrospective cross-sectional study was conducted in the Department of Rheumatology, National Hospital and Medical Centre, Lahore, Pakistan. After receiving consent, electronic medical records (EMRs) of all patients with a diagnosis of noninfectious uveitis (NIU) from November 2019 to January 2023 were reviewed, and a total of 52 patients labeled as having noninfectious uveitis were identified. The collected data included age at diagnosis, anatomical location of uveitis, associated systemic disease, used medications, and outcomes. All cases had been diagnosed and assessed mutually by a rheumatologist and an ophthalmologist using the International Uveitis Study Group classification system to classify the pattern of uveitis by location, clinical course, and laterality and rule out the possibility of other ophthalmologic diseases. Disease activity was defined using the Standardization of Uveitis Nomenclature (SUN) guidelines. Data was analyzed on SPSS Statistics version 23 (IBM Corp, Armonk, NY, USA).

Results: The mean age of the patients in this study was 36.02 ± 43.31 years, with 31 (59.6%) male patients. Anterior uveitis was the most common type observed among the patients at 55.8%, panuveitis was found in 25%, intermediate uveitis and posterior uveitis were seen in 9.6% each. Based on laterality, unilateral eye involvement was identified in 53.8% of patients. Spondyloarthritis (SpA) and idiopathic uveitis were observed in 34.6% and 28.8%, respectively.

In this study, 28 (54.9%) patients were on conventional disease-modifying antirheumatic drugs (cDMARDDs), and 23 (45.1%) were on biological DMARDs. In the biologics group, 82% of patients were in remission in comparison to 60% in the cDMARDs group.

Conclusion: To the best of our knowledge, this is the first report on noninfectious uveitis in the Pakistani population. The study concluded that anterior uveitis is the most common type of uveitis and is more common in males. Spondyloarthropathy is one of the most common underlying systemic diseases. Human leukocyte antigen (HLA)-B27 is associated more with uveitis. Biologics are more effective than cDMARDs in controlling the disease. Collaborative work between different specialties resulted in early diagnosis of underlying systemic disease, better management plans, and disease outcomes. To obtain further details on noninfectious uveitis, a population-based study is needed in Pakistan.

## Introduction

Uveitis refers to a group of intraocular diseases of the uvea, which comprises the iris, ciliary body, and choroid. The adjacent structures are also included such as the retina, optic nerve, cornea, and vitreous humor [1]. Uveitis can further be divided anatomically into anterior, intermediate, posterior, and pan uveitis depending on the extent of inflammation. The etiologic classifications of uveitis are as follows: infectious (bacterial, viral, or fungal causes), noninfectious (known or unknown systemic causes), or masquerade (neoplastic or nonneoplastic origins) [2]. In addition, uveitis is categorized according to the clinical course as 'acute' when it lasts less than three months, 'chronic' when it lasts longer than three months, and 'recurrent' when an acute flare occurs after a previous episode has fully resolved [3].

Noninfectious uveitis (NIU) is an immune-mediated condition characterized by symptoms such as pain, eye floaters, redness of the eye, and light sensitivity [4]. It may also be associated with systemic features of underlying autoimmune disease.

Blindness is one of the worst disabilities caused by NIU. In the United States, uveitis is one of the leading causes of preventable blindness [5] and represents 15% of all conditions that lead to permanent blindness in developed nations [6]. Among patients who are treated for uveitis in academic ophthalmology clinics and referral centers, 20% to 70% experience major vision loss [7]. Up to 50% of patients will experience reduced visual function, and 10% to 15% will become blind [8]. Studies have shown a strong correlation between delayed treatment and the likelihood of poor visual outcomes [9-10]. Uveitis-related vision loss has a detrimental effect on the patient’s daily activities and quality of life [11].

The diagnosis of spondyloarthritis (SpA) or other rheumatic disorders may be made based on the presenting symptoms of uveitis [8]. The most common type of uveitis is acute anterior uveitis, which is frequently linked to spondyloarthropathies, a condition in which uveitis may be the initial sign of the illness. Systemic disorders are typically linked to certain patterns of uveitis [7]. Thus, strong cooperation between ophthalmologists and rheumatologists can prevent the need for pointless diagnostic procedures and is crucial for the accurate diagnosis and treatment of patients [11]. Compared with other types of uveitis, acute anterior uveitis typically has a favorable prognosis [2]. However, frequent occurrences of flares may cause permanent visual loss which can be prevented with close cooperation between the specialties of ophthalmology and rheumatology and timely diagnosis and treatment of patients [3].

Patients who have been referred to a uveitis clinic are usually first seen by an ophthalmologist, who uses the International Uveitis Study Group classification system to classify the pattern of uveitis by location, clinical course, and laterality and rules out the possibility of ophthalmologic diseases [12]. Next, a rheumatologist completes the clinical history and performs the routine diagnostic procedures (chest X-ray, urinalysis, biochemistry, erythrocyte sedimentation rate, treponemal test, and hemogram) for any patient experiencing their first episode of uveitis [11]. Sarcoidosis and syphilis do not have a set pattern, so a treponemal test and chest X-ray are advised [2].

Unfortunately, no extensive data on NIU is available in Pakistan, except for a single study showing the burden of 24% of patients having NIU among a total of 98 uveitis patients [13]. However, in Pakistan, rheumatologists do get referrals from ophthalmology with suspected autoimmune eye diseases to assess systemic diseases and treatment plans. Hence, this study aimed to establish collaborative treatment plans and outcomes and to evaluate the underlying diagnosis of patients suffering from NIU referred to rheumatologists. The secondary objective was to identify the pattern of treatment and outcomes along with the importance of collaborative care and coordination between rheumatologists and ophthalmologists to improve patient care and outcomes.

This article was previously presented as an initial abstract at the Asia-Pacific League of Associations for Rheumatology Congress (APLAR) 24th annual meeting held between December 6 to 9, 2022.

## Materials and methods

This retrospective cross-sectional study was conducted in the Department of Rheumatology, National Hospital and Medical Centre, Lahore, Pakistan, and was approved by its Human Research Ethics Committee (approval no. NHMC/1033). On receiving proper consent, electronic medical records (EMRs) of all patients with a diagnosis of NIU from November 2019 to January 2023 were reviewed, and a total of 52 patients labeled as having NIU were identified. All cases had been diagnosed and assessed mutually by a rheumatologist and an ophthalmologist using the International Uveitis Study Group classification system to classify the pattern of uveitis by location, clinical course, and laterality to rule out the possibility of other ophthalmologic diseases. Disease activity was defined using the Standardization of Uveitis Nomenclature (SUN) guidelines. Underlying systemic disease was diagnosed and labeled based on systemic features, laboratory and radiology findings, and the rheumatologist's clinical evaluation.

The criteria for inclusion were patients with an established diagnosis of NIU based on laboratory results, ophthalmologist's examination findings, and systemic features. The exclusion were all patients who had a diagnosis other than NIU, patients who were lost to follow-up, and patients with incomplete data. Patients who had pre-existing chronic illnesses such as chronic kidney disease, any malignancies, infectious causes of uveitis, and uveitis after trauma were also excluded.

The EMRs of patients tagged with the diagnosis of NIU were reviewed. The data on patient demographics and the type of uveitis, laterality, diagnostic workup, associated systemic disease, treatment plan, and outcomes were recorded. The data regarding conventional disease-modifying antirheumatic drugs (cDMARDs) and biological DMARDs were also retrieved. The outcome or disease activity was based on the rheumatologist and ophthalmologist’s collaborative examination in the most recent clinic visit. Statistical analysis was done using the SPSS Statistic version 23 (IBM Corp., Armonk, NY, USA).

## Results

The present study included 52 patients labeled as having NIU, 31 (59.6%) were male and 21 (40.4%) were female. The patients had a mean age of 36.02 ± 43.31 years, and their ages ranged between 10 and 62 years (Table 1).

**Table 1:** Demographic variables and characteristics of uveitis CCP: Cyclic citrullinated peptides, ANA: Antinuclear antibodies

Demographic variables and characteristics of uveitis	All patients (n=52)
Age (mean SD ±)	36.02 (±43.31)
Male (%)	31 (59.6%)
Anterior uveitis (%)	29 (55.8%
Intermediate uveitis (%)	5 (9.6%)
Posterior uveitis (%)	5 (9.6%)
Panuveitis	13 (25%)
Unilateral uveitis	28 (53.8%)
Bilateral uveitis	24 (46.2%)
Positive rheumatoid factor (%)	1 (1.9%)
Positive anti-CCP levels(%)	2 (3.8%)
Positive ANA (%)	6 (11.5%)

**Figure 1:**
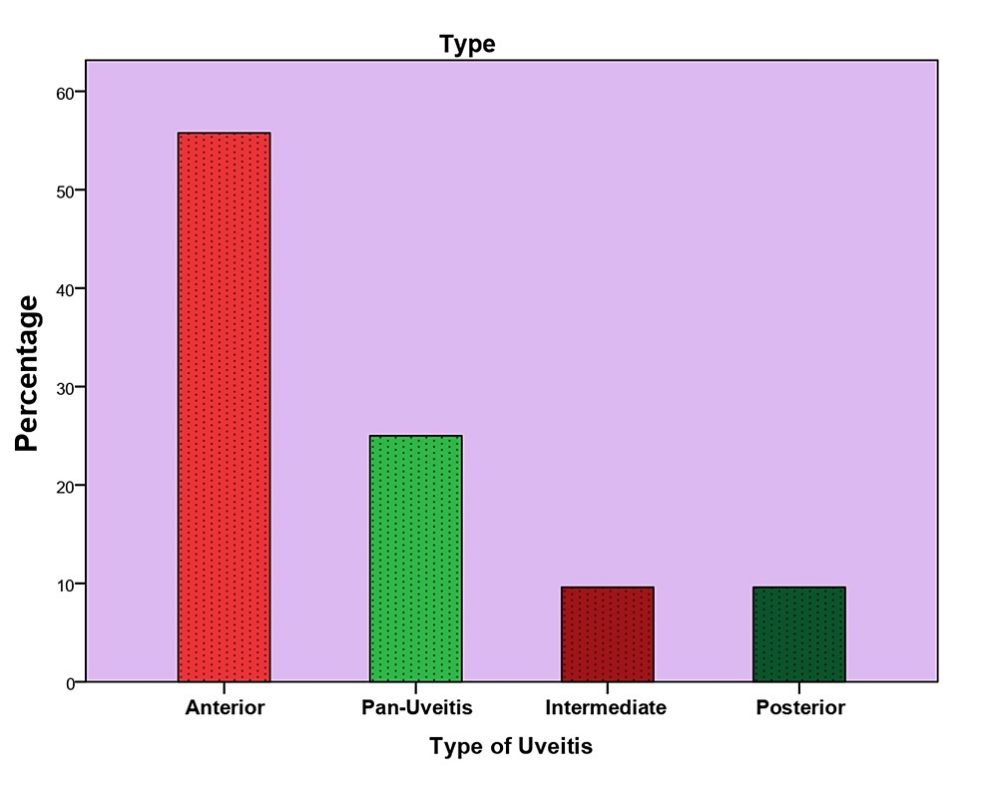
Anatomical distribution of uveitis

Anterior uveitis was the most common type of uveitis observed among the patients at 55.8%. Panuveitis was found in 25% of patients, and both intermediate uveitis and posterior uveitis were found in 9.6% each. Furthermore, unilateral eye involvement was observed in 53.8% of patients, while 7.7% initially had unilateral symptoms and later progressed to bilateral symptoms (Figure 1).

**Figure 2:**
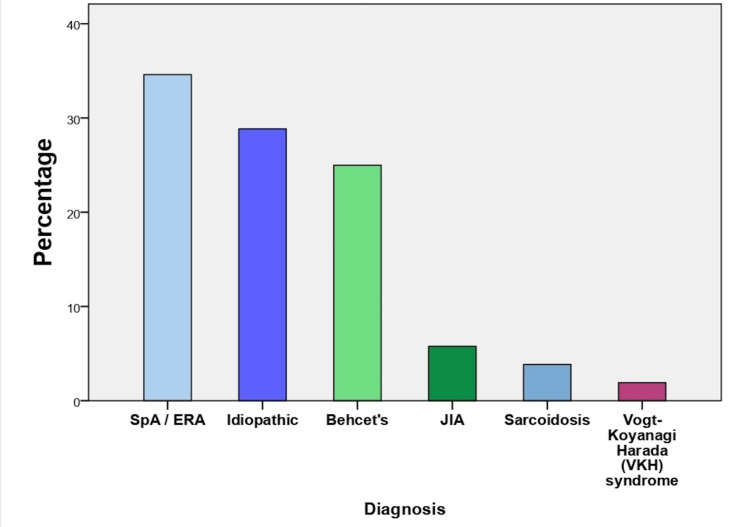
Percentage of underlying disease in patients with NIU SpA: Spondyloarthritis, ERA: Enthesitis-related arthritis, JIA: Juvenile idiopathic arthritis, NIU: Noninfectious uveitis

Regarding underlying diagnosis, spondyloarthropathies including SpA or enthesitis-related arthritis (ERA) were confirmed in 34.6% of patients, predominantly in males at a 2:1 ratio. Idiopathic uveitis was confirmed in 28.8%, Behcet’s disease in 25%, juvenile idiopathic arthritis (JIA) in 5.8%, sarcoidosis in 3.8%, and Vogt-Koyanagi-Harada (VKH) syndrome in 1.9 % patients (Figure 2).

In the present study, out of the 52 participants suffering from uveitis, two (3.8%) had a positive family history of uveitis. The laboratory data indicated that human leukocyte antigen (HLA)-B27 was positive among 15 (28.8%) individuals, and angiotensin-converting enzyme (ACE) levels were raised among two (3.8%) participants. The rheumatoid factor was positive among one (1.9%) patient, and the anti-cyclic citrullinated peptides (CCP) level was positive for two (3.8%) only. Moreover, the antinuclear antibodies (ANA) were positive among six (11.5%) individuals. About 11% of the patients had their first episode of uveitis within six months from the diagnosis of underlying autoimmune systemic disease, while 48.1% had an onset of uveitis within three years of diagnosis of underlying autoimmune disease.

**Figure 3:**
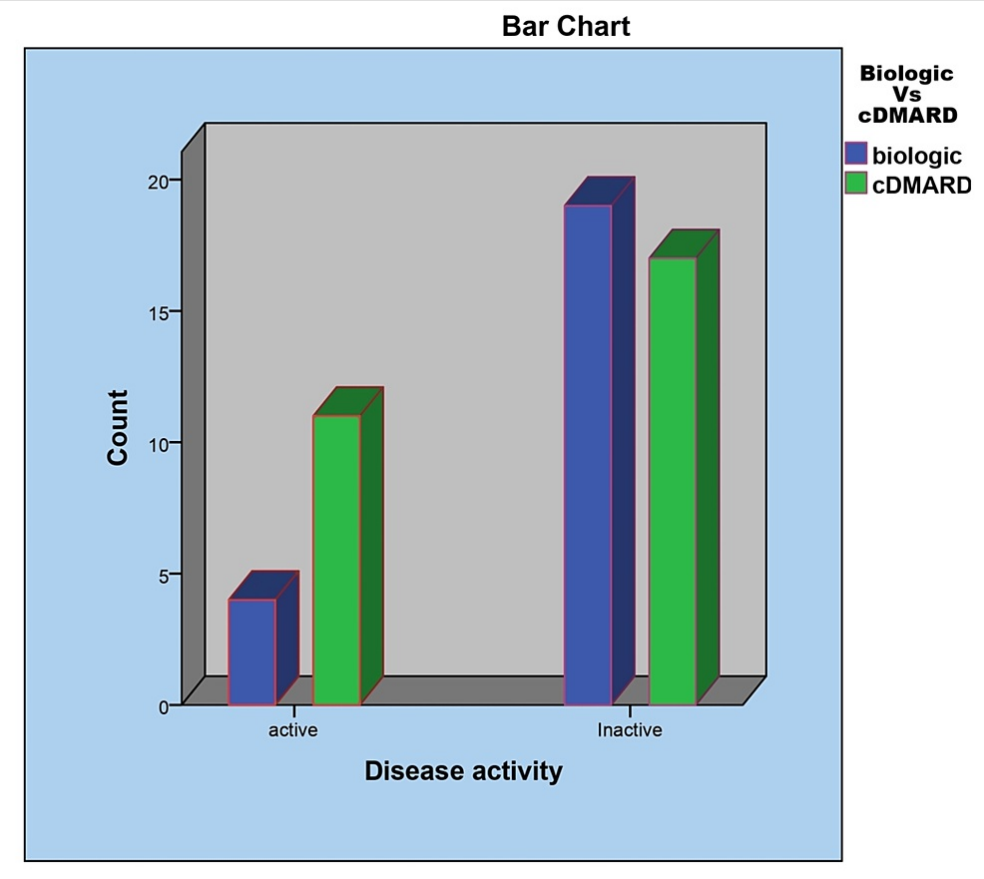
Comparison of treatment outcome in NIU NIU: Noninfectious uveitis

In this study, 28 (54.9%) patients were observed to be on cDMARDs including single drug and in combination with two different drugs from cDMARDs, including methotrexate, cyclosporine, sulfasalazine, and leflunomide, with standard monitoring. Meanwhile, 23 (45.1%) patients were on biological DMARDs including adalimumab 10 (19.2%), infliximab 10 (19.2%), tocilizumab in two (3.9%), and tofacitinib in one patient. Out of the 52 patients, 22 (42.3%) were in remission. It was observed that among the biologics group, 19 out of the 23 (82%) patients were in remission in comparison to 17 out of 28 (60%) in the cDMARDs group (Figure 3).

## Discussion

Uveitis is a disease with different presentations and etiologies. For optimal and prompt management, it is mandatory to reach a probable underlying diagnosis that is influenced by clinical signs in a patient to make decisions regarding treatment. Infections remain the most common cause of uveitis, as evidenced by many studies worldwide and in Pakistan, but NIU is always difficult to diagnose and treat early due to delays in diagnosis and referrals to rheumatologists. Strong collaboration between departments can be helpful in early diagnosis and treatment, enabling better outcomes and higher remission rates of the disease.

Ophthalmologists should at the very least refer all patients with NIU to a rheumatologist for diagnostic purposes when a multidisciplinary uveitis clinic is not practical. In the ideal scenario, patients with any non-ophthalmologic uveitis should undergo a thorough clinical examination by a rheumatologist [3]. A researcher demonstrated the diagnosis and new emerging techniques in treating NIU. The findings evaluated that biologics, Janus kinase and signal transducer and activator of transcription (JAK-STAT) inhibitors, and improved localized therapies may provide additional options for patients with NIU. Moreover, laboratory findings such as blood tests and various autoimmune tests are effective in diagnosing NIU [11].

Another research was conducted to determine uveitis care outcomes in a standalone versus a combined ophthalmology-rheumatology clinic. The study recruited patients who were aged 18 years and above and had a minimum chronic uveitis history of 12 months. The findings of the study revealed that combined care by rheumatologists and ophthalmologists leads to better outcomes and improvement among patients [2].

In this study, male patients are more affected than female patients, whereas epidemiological surveys of different studies showed variation in different geographical regions where women are more affected than men [2]. The laboratory data indicated blood tests such as ACE levels, rheumatoid factor, anti-CCP level, cytoplasmic-antineutrophil cytoplasmic autoantibody (C-ANCA) level, and ANA status appeared to have a maximum of negative results among patients suffering from uveitis. However, HLA-B27 appeared to be different, with a maximum number of positive results. The study indicated anterior uveitis as the most common type of uveitis.

Jack et al. demonstrated the role of laboratory findings in diagnosing anterior uveitis [3]. The authors recruited 39 participants aged 10 to 20 years. The laboratory findings such as HLA-B27, rheumatoid factor, and ANA were evaluated. Patients with uveitis were more often male, HLA-B27-positive, and younger. The findings of the present study indicated that HLA-B27 was more specifically associated with spondyloarthritis and with uveitis. The sensitivity of the test was 94% [3]. 

Another study conducted by Khan et al. evaluated the diagnostic value of laboratory workup. The findings of the study demonstrated that laboratory workup holds a potential advantage in diagnosing uveitis. Among all the laboratory workups, HLA-B27 is effective in diagnosing the disease [12]. The results are similar to the findings of this study. In contrast, Murat et al. evaluated diagnostic testing in uveitis. Infectious uveitis and NIU were diagnosed using laboratory findings. The findings indicated that ANA and rheumatoid factor is also effective in diagnosing underlying systemic disease with manifestations of uveitis [14].

In the present study, most of the patients on cDMARDs were tolerant to therapy, and methotrexate was one of the most common cDMARDs used in patients. Some patients were on a combination of cDMARDs, including methotrexate plus leflunomide, methotrexate plus cyclosporine, and sulfasalazine plus methotrexate with standard monitoring.

The findings of this study revealed that biologics are more effective in treating uveitis compared to cDMARDs. A study conducted by Ming et al. [15] evaluated the use of biological therapies in uveitis. The findings of the study revealed that adalimumab appears to have promising results in treating autoimmune-mediated uveitis, whereas infliximab is effective in treating Behcet’s disease. The different biologics have variable effectiveness in different autoimmune diseases because of their different targets on inflammatory pathways. These findings [15] are in accordance with the findings of our study.

Imrie et al. concluded that biological therapies are effective in treating uveitis. Moreover, they expanded the treatment options for sight-threatening uveitis [16]. These findings too, are in accordance with the findings of our study. In contrast, Heo et al. demonstrated that biologics should not be used as the first line of therapy in treating uveitis. This is due to high cost, inconvenience, and immunosuppressive effects [17].

Jacqueline et al. demonstrated the most common types of uveitis in patients with juvenile inflammatory rheumatic diseases [18]. The study concluded that anterior uveitis is the most common type of uveitis. Their findings are similar to the findings of the present study.

This study holds the limitation of a smaller sample size. Moreover, the causality of a relationship cannot be determined. Another limitation of our study is its retrospective nature. Furthermore, data was recorded as per most recent visits in clinics from EMRs and not directly from patients. Also, it was conducted in a single center rather than being population-based, which would have given more details about NIU.

## Conclusions

Anterior uveitis is the most common type of uveitis and is more common in males. Spondyloarthropathy is one of the most common underlying diseases. The HLA-B27 is closely associated with uveitis. Biologics are more effective than cDMARDs in controlling the disease. Collaborative work between different specialties resulted in early diagnosis of underlying systemic disease, better management plans, and disease outcomes.

## References

[1] Rosenbaum JT, Bodaghi B, Couto C (2019). New observations and emerging ideas in diagnosis and management of non-infectious uveitis: a review. Semin Arthritis Rheum.

[2] Ross BX, Habhab S, Syeda S (2022). Patient clinical outcomes in standalone versus a combined ophthalmology-rheumatology uveitis clinic. J Ophthalmic Inflamm Infect.

[3] Walscheid K, Glandorf K, Rothaus K, Niewerth M, Klotsche J, Minden K, Heiligenhaus A (2021). Enthesitis-related arthritis: prevalence and complications of associated uveitis in children and adolescents from a population-based nationwide study in Germany. J Rheumatol.

[4] Dick AD, Rosenbaum JT, Al-Dhibi HA (2018). Guidance on noncorticosteroid systemic immunomodulatory therapy in noninfectious uveitis: fundamentals of care for uveitis (FOCUS) initiative. Ophthalmology.

[5] Touhami S, Diwo E, Sève P (2019). Expert opinion on the use of biological therapy in non-infectious uveitis. Expert Opin Biol Ther.

[6] Hsu YR, Huang JC, Tao Y (2019). Noninfectious uveitis in the Asia-Pacific region. Eye (Lond).

[7] Marino A, Weiss PF, Davidson SL, Lerman MA (2019). Symptoms in noninfectious uveitis in a pediatric cohort: initial presentation versus recurrences. J AAPOS.

[8] Chateau T, Angioi K, Peyrin-Biroulet L (2020). Two cases of successful ustekinumab treatment for non-infectious uveitis associated with Crohn's disease. J Crohns Colitis.

[9] Kaburaki T, Fukunaga H, Tanaka R (2020). Retinal vascular inflammatory and occlusive changes in infectious and non-infectious uveitis. Jpn J Ophthalmol.

[10] Schnabel A, Unger E, Brück N (2020). High-dose intravenous methylprednisolone in juvenile non-infectious uveitis: a retrospective analysis. Clin Immunol.

[11] Takeuchi M, Mizuki N, Ohno S (2021). Pathogenesis of non-infectious uveitis elucidated by recent genetic findings. Front Immunol.

[12] Khan A, McGuffey CD, Melson AT, Riaz KM (2022). Acute anterior uveitis with HLA-B27 positivity after corneal cross-linking with previous intrastromal corneal ring segment. Case Rep Ophthalmol Med.

[13] Ishaq M, Muhammad JS, Mahmood K (2012). Uveitis is not just an ophthalmologists' concern. J Pak Med Assoc.

[14] Hasanreisoglu M, Kesim C (2020). Diagnostic testing in uveitis. J Retin-Vitr.

[15] Ming S, Xie K, He H, Li Y, Lei B (2018). Efficacy and safety of adalimumab in the treatment of non-infectious uveitis: a meta-analysis and systematic review. Drug Des Devel Ther.

[16] Li B, Yang L, Bai F, Tong B, Liu X (2022). Indications and effects of biological agents in the treatment of noninfectious uveitis. Immunotherapy.

[17] Thomas AS (2019). Biologics for the treatment of noninfectious uveitis: current concepts and emerging therapeutics. Curr Opin Ophthalmol.

[18] Hayworth JL, Turk MA, Nevskaya T, Pope JE (2019). The frequency of uveitis in patients with juvenile inflammatory rheumatic diseases. Joint Bone Spine.

